# Mechanical and Water Uptake Properties of Epoxy Nanocomposites with Surfactant-Modified Functionalized Multiwalled Carbon Nanotubes

**DOI:** 10.3390/nano11051234

**Published:** 2021-05-07

**Authors:** Arya Uthaman, Hiran Mayookh Lal, Chenggao Li, Guijun Xian, Sabu Thomas

**Affiliations:** 1 Key Lab of Structures Dynamic Behavior and Control, Harbin Institute of Technology, Ministry of Education, Heilongjiang, Harbin 150090, China; aryauthaman@yahoo.co.in (A.U.); hiran009@yahoo.co.in (H.M.L.); gjxian@hit.edu.cn (G.X.); 2 Key Lab of Smart Prevention and Mitigation of Civil Engineering Disasters of the Ministry of Industry and Information Technology, Harbin Institute of Technology, Harbin 150090, China; 3 School of Civil Engineering, Harbin Institute of Technology, Heilongjiang, Harbin 150090, China; 4 School of Energy Materials, Mahatma Gandhi University, Kerala 686560, India; sabuthomas@mgu.ac.in

**Keywords:** carbon nanotubes (CNTs), nanocomposites, epoxy resin, mechanical and thermal properties, water uptake

## Abstract

The superior mechanical properties of multi-walled carbon nanotubes (MWCNTs) play a significant role in the improvement of the mechanical and thermal stability of an epoxy matrix. However, the agglomeration of carbon nanotubes (CNTs) in the epoxy is a common challenge and should be resolved to achieve the desired enhancement effect. The present paper investigated the thermal, mechanical, and water uptake properties of epoxy nanocomposites with surfactant-modified MWCNTs. The nanocomposites were prepared through the incorporation of different weight concentrations of MWCNTs into the epoxy matrix. Comparative analysis of neat epoxy and epoxy/CNT nanocomposites were conducted through thermal, mechanical, microscopic, and water uptake tests to reveal the improvement mechanism. The homogenous distribution of the CNTs in the epoxy was achieved by wrapping the surfactant onto the CNTs. The addition of surfactant-modified CNTs into the epoxy caused an obvious increase in the mechanical and thermal properties. This improvement mechanism could be attributed to the uniform dispersion of the CNTs in the epoxy matrix reducing the free volume between the polymer chains and restricting the chain segmental mobility, leading to strong interfacial bonding and an efficient load transfer capability between the CNTs and the epoxy matrix. However, the mechanical and thermal properties of the epoxy/CNT nanocomposite decreased owing to the agglomeration effect when the concentration of the CNTs exceeded the optimal percentage of 1.5%. Additionally, the CNTs could impart a reduction in the wettability of the surface of the epoxy/CNT nanocomposite, leading to the increase in the contact angle and a reduction in the water uptake, which was significant to improve the durability of the epoxy. Moreover, the higher weight concentration (2%) of the CNTs showed a greater water uptake owing to agglomeration, which may cause the formation of plenty of microcracks and microvoids in the nanocomposite.

## 1. Introduction

Considering Lijima’s report from 1991 and onwards, CNTs get much attention in science and engineering. The extraordinary physical, chemical, and mechanical properties of CNTs give them application potential in many fields. CNTs are excellent fillers due to their high strength and significant role in the nanocomposites used in civil engineering and aerospace applications [1]. There are several types of CNTs, such as single-walled, double-walled, and multi-walled CNTs. The multi-walled CNTs (MWCNTs) possess the advantages of low cost and easy dispersion, and can remarkably improve the mechanical properties of nanocomposites [2]. The main limitation of the single-walled CNTs over other CNTs is the difficultly to disperse them, even though they have an efficient load transfer [3]. CNTs can be added to polymers to improve the mechanical and thermal properties of engineering polymers, such as epoxy resin [4,5]. Generally, regarding the several types of epoxies available, amine-cured epoxies are considered for the polymer matrix in fiber reinforced composites due to their superior engineering performance [6,7]. Allaoui et al. reported that the addition of CNTs enhanced the modulus and strength of the epoxy matrix [8]. The effective interface load transfer between the CNTs and the matrix creates the higher mechanical properties of the epoxy matrix [9,10]. 

Added to the benefits of CNTs, the dispersion of CNTs in the epoxy matrix is still a challenge. Since CNTs tend to agglomerate due to their weak intermolecular interactions like Van der Waals forces, their pi–pi interactions between the pi clouds of the aromatic rings [11], their hydrophobic interaction, and their ease in forming as bundles, which lead to weak interface bonding between the polymer matrix and the CNTs. Some studies revealed that functionalized CNTs helped to reduce the above agglomeration phenomenon. Compared with other interactions, the interphase binding force between the epoxy matrix and CNTs grafted with –OH and –COOH groups are primarily by the weak Van der Waals force. When it comes to CNTs grafted with NH_2_ groups, both the hydrogen and covalent bonds are supposed to form between the NH_2_-CNTs and the epoxy matrix. Therefore, the binding force of the interphase can be greatly enhanced. However, the agglomeration of CNTs is still a challenge for developing nanocomposites with a high performance for engineering applications.

While mixing CNTs into the polymer matrix, the uniform distribution of nanoparticles must be ensured without destroying the integrity of the particles. Generally, the modification of CNTs is one of the best methods for preventing the agglomeration of CNTs. Modification of CNTs can be composed mainly of two types: covalent bonding and non-covalent bonding. Regarding the non-covalent bonding, CNTs are wrapped or adsorbed onto the surface of polymers, while covalent bonding can be realized through a chemical interaction [12]. Hence, many researchers adopt various methods to improve the dispersion state, such as ultrasonication, mechanical mixing, surfactant adding, chemical functionalization, and polymer wrapping [13,14]. Furthermore, the adding of surfactants is effective for reducing the agglomeration of CNTs in the polymer.

There are many varieties of surfactants available, commonly in which Polyvinyl Pyrrolidone (PVP) is the most effective surfactant due to its unique properties. They were chosen here as the organic components to realize the uniform dispersion of nanoparticles in the polymer. PVPs are soluble in polar solvents (such as alcohol) and can enhance the thermal and mechanical properties of the filler and polymer by increasing the cross-linking between the molecular chains. Moreover, the amorphous structure of PVPs decrease the scattering loss and also can be used in optical applications [15]. PVP consists of hydrophilic constituents, such as the pyrrolidone moiety (C=O, C–N) and hydrophobic groups, such as the alkyl (CH_2_). The presence of the polar amide group in the pyrrolidone ring and the polar methylene and methine groups can produce a strong steric influence and can control the CNTs agglomeration through the repulsive force from its hydrophobic carbon chains [16].

There are different methods to introduce the CNTs into the epoxy matrix, such as high-speed shear mixing, mechanical mixing, sonication, etc. Montazeri and Chitsazzadeh observed that the ultrasonication process can improve the dispersion of the CNTs and increase the strength and modulus of the epoxy/CNT nanocomposites [17]. Here, the ultrasonication method was adopted and we observed that agglomeration of the CNTs occurred after the dispersion. Therefore, here PVPs have been used as the surfactant for the CNTs, to improve the CNT dispersion in epoxy. The surfactant PVP can wrap onto the surface of the CNTs with a strong force, prevent agglomeration, and improve the thermal and mechanical properties of the nanocomposite. Moreover, the thermal analysis, mechanical properties, and morphological analyses of the epoxy/CNT nanocomposites were conducted to reveal the improvement mechanism. Finally, the effect of the CNTs on the water uptake of epoxy was investigated. 

## 2. Materials and Methods

### 2.1. Raw Materials 

**Figure 1:**
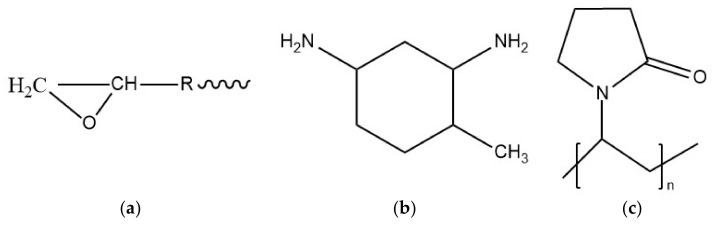
The chemical structures of (**a**) E51 epoxy, (**b**) HTDA, and (**c**) PVP.

The commercially available diglycidyl ether of bisphenol-A (DGEBA)-based E51 epoxy was adopted in the present paper and 4-methyl-1,3 cyclohexane diamine (HTDA, C_7_H_16_N_2_) was selected as the hardener as supplied by Sinopec Shanghai Petrochemical Company (Shanghai, China). The functionalized MWCNT-NH_2,_ with the size of 50 μm (length) and 8–15 nm (outer diameter), was adopted through the chemical vapor deposition (CVD) method with a purity of 95 wt.%. Polyvinyl Pyrrolidone (PVP) was selected as the surfactant for dispersing the MWCNT and 99.9% denatured ethanol from Tianli Chemical Reagent Co. Ltd., (Tianjin, China) was adopted as the dispersing media or solvent. The chemical structure of the epoxy, hardener, and surfactant PVP are shown in Figure 1.

### 2.2. Methods

#### 2.2.1. Preparation of Neat Epoxy Specimens

Neat epoxy matrix specimens were prepared by mixing the E51 epoxy into the HTDA hardener using a vacuum oven to remove air bubbles. Then, the mixture was poured into a mold, kept at room temperature, and post cured in an oven at 120 °C for 2 h. 

#### 2.2.2. Preparation of Epoxy/CNT Nanocomposites

**Figure 2:**
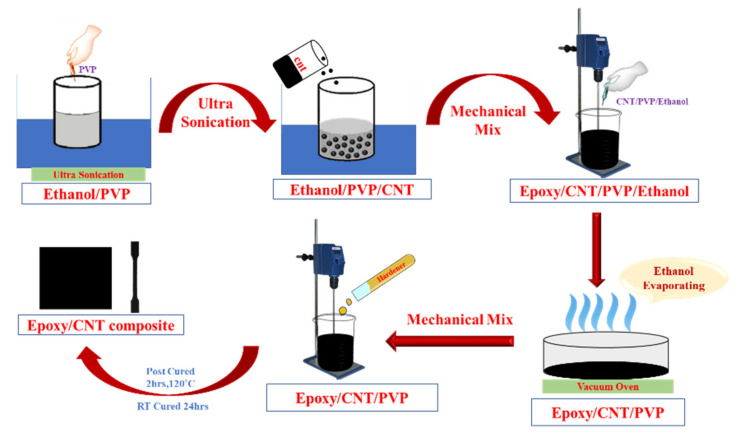
Preparation schematic of the Epoxy/CNT nanocomposites.

**Figure 3:**
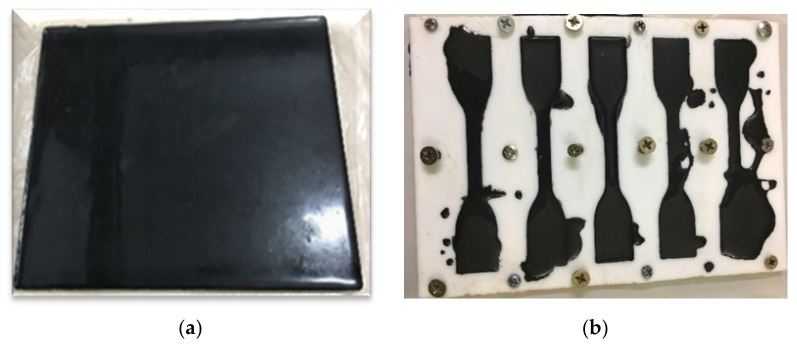
Photographic images of (**a**) Cured epoxy/CNT nanocomposite and (**b**) Uncured epoxy/CNT nanocomposite for the tensile tests.

The MWCNTs were added into the PVP/ethanol solution and sonicated in an ultra-sonication bath. The preheated (50 °C) epoxy matrix was weighed and put into a beaker. Then, the CNT/PVP/ethanol solution was added slowly into the epoxy resin and blended using a mechanical mixer at the speed of 3000 rpm for 1 h. To evaporate the ethanol, the mixture was kept in a vacuum oven below the melting temperature of ethanol. After the ethanol was evaporated completely, the hardener was added into the mixture and stirred at the speed of 150 rpm for 10 min. The air bubbles were removed by placing the mixture into the vacuum chamber. The bubble-free mixture was then poured into a mold, cured at room temperature for 24 h, and post cured at 120 °C for 2 h. The schematic representation of the preparation procedure of the epoxy/CNT nanocomposite is shown in Figure 2. The weight concentrations of the CNTs in the nanocomposite ranged from 0 to 2.0% in 0.5% increments and were prepared and tested. Figure 3 represents the photographic image of the epoxy/CNT nanocomposites.

### 2.3. CNT Dispersion with PVP 

**Figure 4:**
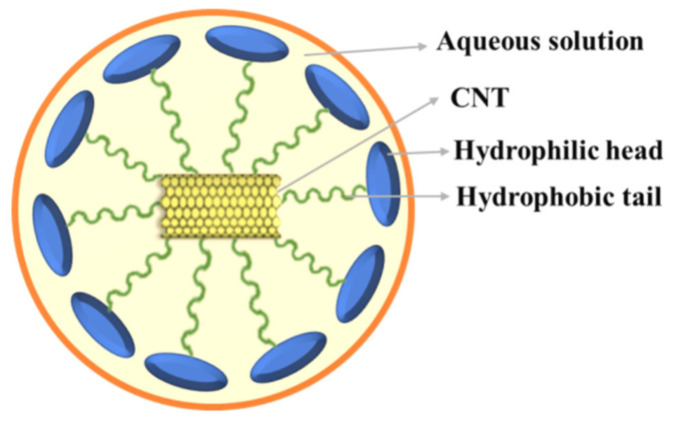
Wrapping process of PVP on the surface of CNTs.

Owing to the strong Van der Waals intermolecular interaction, the CNTs were found to be bundle- or rope-like structures. Moreover, the CNTs could again combine and form tangled networks or agglomerates with lower mechanical properties than the individual CNTs. Researchers were seeking the methods to achieve a better dispersion of CNTs in various solvents using surfactants, such as water-soluble polymers and ionic and non-ionic solvents. To prevent agglomeration, the surfactants (such as PVP) were observed to develop micelles around the CNTs [18,19,20,21,22]. The binding mechanism of the CNTs with PVP (as a surfactant) is illustrated in Figure 4.

**Figure 5:**
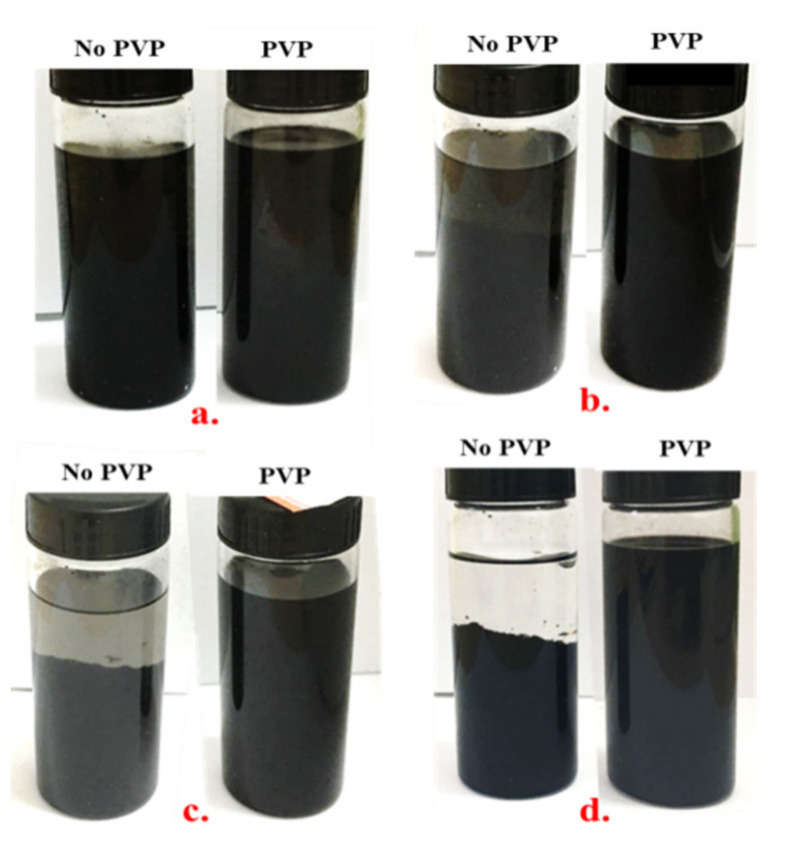
CNTs with and without PVP dispersion in ethanol with the time of (**a**) 0 min, (**b**) 30 min, (**c**) 1 h and (**d**) 3 months.

The dispersion of the CNTs in the epoxy matrix was achieved through the wrapping of the sidewalls onto the surface of the CNTs. Generally, the surfactants interacted with carbon via the hydrophobic segments, meanwhile the hydrophilic segments reacted with the epoxy via hydrogen bonding [23]. Therefore, the non-covalent surfactant (PVP) was chosen for the present study. PVP could be wrapped onto the surface of the hydrophobic CNTs with their hydrophobic tail, through a thermodynamically driven wrapping process. The CNT dispersion in 99.9% denatured ethanol solution with and without PVP was comparatively studied. The stability of the CNT/PVP solution at different time intervals is shown in Figure 5. It can be seen that no or less agglomeration occurred in the PVP solution, indicating that PVP can be used as an efficient surfactant for the dispersion of CNTs.

### 2.4. Mechanical Properties Tests

The flexural tests of the epoxy/CNT nanocomposites were conducted with a universal testing machine (DHY-10080, Hengyi Precision Instrument Co. Ltd., Shanghai, China) according to the standards ASTM D790. The ratio of the span length to the thickness of the specimens was 16:1. The test parameters included the support span and maximum deflection. The loading rate was set as 2 mm/min. Five repeated samples were tested and the average result was obtained for each weight percentage of the CNTs.

The tensile tests of the specimens were conducted to obtain the tensile strength, tensile modulus, and elongation at break. The dumbbell shaped samples were prepared and tested using a universal testing machine (DHY-10080, Hengyi Precision Instrument Co. Ltd., Shanghai, China) based on ASTM D638. The extensometer of the gauge length of 10 mm was adopted to obtain the strain variation during loading. The loading rate was 1 mm/min. To obtain accurate results, five samples of each condition were tested, and the average was recorded.

### 2.5. Dynamic Mechanical Analysis (DMA)

Dynamic mechanical analysis represents an effective non-destructive technique to investigate the viscoelastic characteristics of polymeric materials, such as the storage modulus, loss modulus, and tan delta. The tests were conducted using the Dynamic Mechanical Analyzer (Q800, TA Instruments Co., New Castle, DE, USA). The dual cantilever mode was adopted for a rectangular specimen. Temperature scans from 20 °C to 250 °C at a 1 Hz frequency strain mode with a heating rate of 5 °C/min were performed.

### 2.6. Morphological Analysis

The surface morphology analysis of the specimens was conducted using scanning electron microscopy (Merlin compact with Gemini-I electron column, Zeisis Pvt. Ltd., Jena, Germany) at room temperature. Before the tests, all samples were coated with gold by a Gatan Model 682 precision etching coating system for about 15 min to improve the electron conductivity of the samples. The cryogenic fracture surface analysis was examined at a voltage of 10.0 kV.

The transmission electron microscopy of the specimens was examined through JOEL JEM 2100. The specimens were prepared by dispersing the CNTs into the ethanol solution with and without PVP. A single drop of this dispersion from both specimens was placed on a carbon-coated copper grid. Then the specimens were dried at room temperature.

### 2.7. Contact Angle Tests

The contact angle tests of the samples were conducted at room temperature using a goniometer (Drop Meter TM Element A-60, Maist, Ningbo, China). The contact angle test was realized by placing a droplet of a liquid at various spots on the sample surface and recording the images. The image acquisition and the determination of the contact angle were obtained using Drop Meter Software. More than ten readings of each sample were taken, and the average was obtained.

### 2.8. Water Uptake Tests

Water uptake tests were performed through placing the specimens into water for 60 days at room temperature in accordance with ASTM D570. The weight of the samples was recorded before the immersion in deionized water at room temperature. The weighing of the samples at regular intervals used a high-precision four-digit electronic balance. 

## 3. Results and Discussion

### 3.1. Mechanical Properties

#### 3.1.1. Flexural Properties

**Figure 6:**
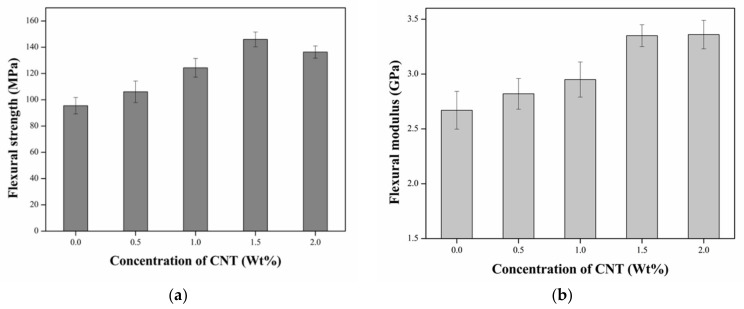
Dependence of flexural properties of epoxy/CNT nanocomposites on the weight concentration of CNTs of (**a**) flexural strength and (**b**) flexural modulus.

Figure 6 shows the dependence of the flexural properties of the epoxy/CNT nanocomposites on the concentration of the CNTs. Table 1 summarizes the increase in the percentage of the flexural strength and modulus. Shown in Figure 6a, the addition of CNTs into the epoxy matrix can improve the flexural strength and modulus of the nanocomposites. The flexural strength of the neat epoxy matrix was 95.46 MPa and was enhanced by the increase in the CNTs. The increase percentages of the flexural strength of the epoxy/CNT composites were 11.1%, 30.3%, 52.9%, and 42.8% when the added weight concentrations of the CNTs were 0.5%, 1%, 1.5%, and 2%, respectively. Additionally, the nanocomposites achieved the maximum flexural strength (146 MPa) when the weight concentrations of the CNTs were 1.5%, which indicated strong interfacial bonding facilitated the efficient load transfer between the epoxy and the CNTs, resulting in an upsurge in flexural strength. It was noticed that the flexural strength decreased when the weight concentration of the CNTs was more than 1.5%, because the agglomeration of the CNTs occurred at higher concentrations since the increase in the resin viscosity caused a consequent resistance to dispersion. 

Figure 6b shows the flexural modulus vs. the weight concentration of the CNTs. The increase in the percentage of the flexural modulus of the nanocomposites was more than 25% compared to the neat epoxy matrix. Furthermore, the percentage increases in the flexural modulus of the epoxy/CNT nanocomposites were 5.6%, 10.5%, 25.5%, and 25.8% when the added weight concentrations of the CNTs were 0.5%, 1%, 1.5%, and 2%, respectively. The maximum or equivalent increments in the flexural modulus were observed when the weight concentrations of the CNTs were 1.5% and 2%. The above obvious increase in the flexural modulus was attributed to the reinforcing effect of the CNTs on the epoxy matrix.

Based on the above analysis, the increment of the flexural strength and modulus mainly depended on the added content of the CNTs. The flexural strength and modulus were augmented remarkably with the increase in CNTs. However, the saturation or reduction in the flexural properties occurred when the content of the CNTs exceeded the optimal content. This was because the dispersion of moderate CNTs in the epoxy matrix may boost the flexural properties [24]. Excessive content of the CNTs could lead to agglomeration since the attractive forces formed from pi electrons on the surfaces lead to a decrease in the flexural properties [25]. Additionally, the incorporation of the CNTs into the epoxy matrix led to a higher elongation at break, indicating an obvious toughening effect. 

#### 3.1.2. Tensile Properties

**Figure 7:**
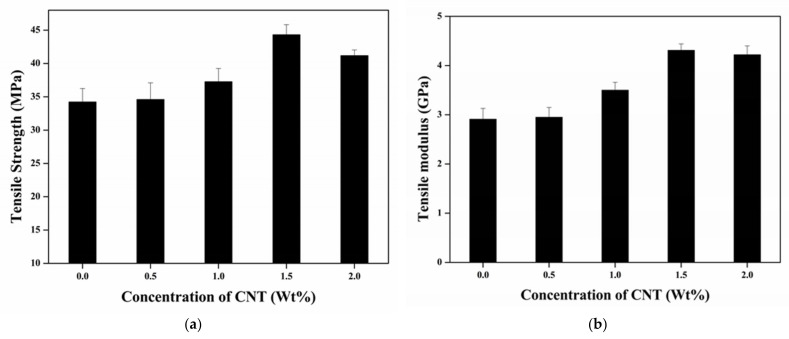
Dependence of tensile properties of epoxy/CNT nanocomposites on the weight concentration of CNTs of (**a**) tensile strength and (**b**) tensile modulus.

Figure 7 represents the dependence of the tensile properties of the epoxy/CNT nanocomposites on the weight concentration of the CNTs. Similar to the flexural properties, the addition of CNTs into the epoxy matrix augmented the tensile strength and modulus of the nanocomposites. Moreover, the percentage increases in the tensile strength of the epoxy/CNT nanocomposites were 1.1%, 8.9%, 29.5%, and 20.3% when the supplemented weight concentrations of the CNTs were 0.5%, 1%, 1.5%, and 2%, respectively. The percentage increases in the tensile modulus of the epoxy/CNT nanocomposites were 1.4%, 20.3%, 48.1%, and 45.0%, respectively. Table 2 lists the increased percentages of the tensile strength and tensile modulus accompanying the increase in the CNTs. It can be observed that the optimal percentage of added CNTs was 1.5% to obtain the maximum increase in the tensile strength and modulus. The enhancement mechanism of the tensile strength of the nanocomposites was attributed to the increase in the interfacial bonding between the epoxy matrix and the CNTs, owing to the formation of covalent bonds. A similar phenomenon was shown in the study on the improvement of the tensile strength through the incorporation of carboxyl functionalized CNTs, rather than pure CNTs [26]. 

Additionally, the increase in the tensile modulus of the epoxy/CNT nanocomposites was ascribed to the strong bonding of the functional group formed between the CNTs and the epoxy matrix which restricted the mobility of the main chain of the epoxy matrix. Similar improvement accompanying the increase in CNT concentration was observed in the study [24]. Furthermore, the agglomeration of the CNTs in the epoxy matrix occurred when the CNT concentration was 2.0% due to the non-uniform dispersion of the CNTs, since the higher concentration of the nanoparticles led to the poor load transfer between the CNTs and the epoxy [27]. Hence, the effect of CNT content on the tensile properties of the epoxy matrix was significant. The excessive CNT content may affect the dispersion and polymerization reactions [28,29,30], leading to a reduction in the tensile properties. 

According to the above analysis, the tensile strength and modulus enlarged with the incorporation of the CNTs in the epoxy. A similar improvement effect on the tensile properties was summarized as follows. Montazeri et al. [31] observed that the tensile strength and modulus increased until at the maximum weight percentage (2.0%) of the CNTs. According to Zhou et al. [32], the addition of pristine CNTs led to a reduction in the tensile strength and modulus of the nanocomposites. Montazeri et al. [17] reported that the tensile strength and modulus of the epoxy/CNT nanocomposites improved when the CNT concentration was not more than 3 wt.%. According to Srivastava et al. [33], the mechanical properties, such as the tensile and compressive strengths, increased with the concentration of the CNTs owing to the formation of network structures of MWCNTs in the epoxy matrix and, to a greater extent, it could take the mechanical stress applied to the nanocomposites. Moreover, other researchers reported that the uniform distribution of the CNTs in epoxy led to an increase in the strength and modulus, and a reduction in the elongation at break [34]. Therefore, the content of the CNTs played a significant role in the tensile properties of the epoxy nanocomposite. An optimal added content of CNT concentration was determined to be 1.5%, in the present paper, to achieve the maximum improvement in the tensile properties.

### 3.2. Dynamic Mechanical Analysis (DMA)

**Figure 8:**
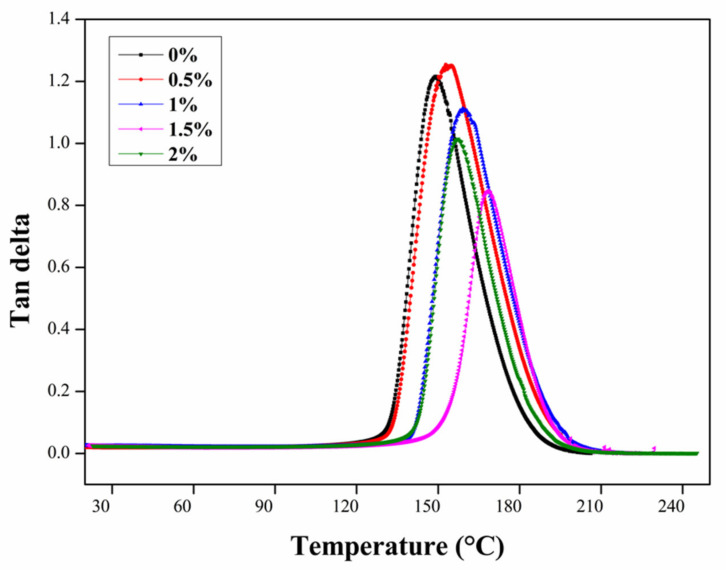
Tan delta vs. temperature of the epoxy/CNT nanocomposites.

Figure 8 shows the tan delta vs. temperature curves of the epoxy/CNT nanocomposites at different weight concentrations of CNTs. The thermal endurance properties of the epoxy matrix obviously improved through the addition of the CNTs. The maximum increase percentage (~9.5%) of the glass transition temperature (Tg) of the nanocomposites, for example, was achieved for the optimal added ratio (1.5%) of the CNTs compared to the neat epoxy matrix. However, the higher added ratio (2%) of the CNTs caused a decrease in the Tg, which was attributed to the agglomeration of the CNTs in the epoxy acting as a plasticizer and decreasing the crosslinking and interfacial bonding between the epoxy matrix and the CNTs in the nanocomposite [35]. Furthermore, the interfacial reaction diminished the mobility of the epoxy matrix [36]. A similar phenomenon was observed in the following investigations. Gong et al. [37] reported that the CNTs can make an increase in the Tg and storage modulus in a nanocomposite. Additionally, the surfactant also can change or reduce the modulus, since the surfactants can behave as a plasticizer. According to Armstrong [35], if the CNTs are not covalently bonded with the epoxy matrix, then the CNT particles may act as a plasticizer and reduce the glass transition temperature. Khare et al. [38] reported that the epoxy nanocomposite with dispersed pristine CNTs showed a depression in the glass transition temperature (Tg), since the weak interaction between the matrix and nanofiller resulted in the reduction of the Tg compared to the neat polymer. Thus, a decrease in the thermal properties by the addition of CNTs also was reported. However, the present study, found that the surfactant-modified functionalized CNTs could be dispersed uniformly in the epoxy matrix, which enhanced the interfacial interaction and mechanical properties of the epoxy nanocomposites.

Additionally, it can be observed that the peak of the tan delta curves of the nanocomposites gradually decreased with the added content of the CNTs and reached the minimum at the optimal addition ratio of the CNTs. The tan delta represents the energy dissipation of materials and the lower peak of the tan delta indicates the better interfacial bonding between the CNTs and the matrix. Furthermore, by introducing CNTs into the epoxy, the rise of the glass transition temperature occurred due to the higher crosslinking density. Also, this can be ascribed to the free space reduction happening in the macromolecules or the reduction in the molecular motion within the polymer chain segments.

### 3.3. Morphological Analysis

**Figure 9:**
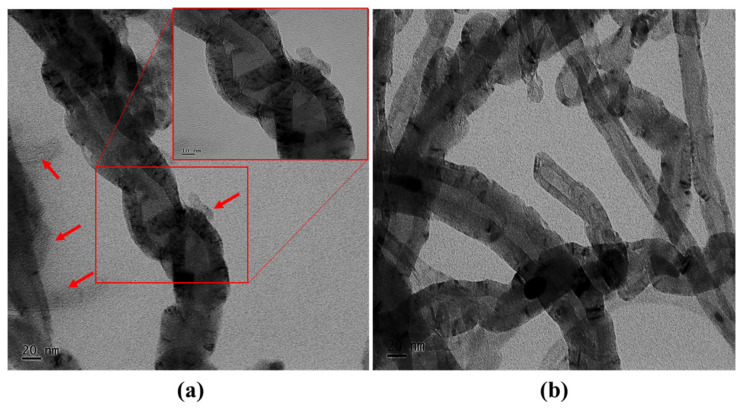
TEM images of (**a**) PVP coated CNTs and (**b**) CNTs.

Figure 9 represents the transmission electron microscopic (TEM) images of the PVP-coated CNTs and uncoated CNTs. The arrows indicated in Figure 9a are considered to be PVP, and the squared area represents the coated PVP on the surface of the CNTs, which is shown as an amplified image (10 nm). A thin coating of PVP on the surface of the CNTs was observed, which had an obvious effect on the mechanical, thermal, and water uptake properties of the epoxy/CNT nanocomposites by inhibiting the agglomeration of the CNTs in the epoxy matrix. Additionally, from the morphological analysis, the uniform dispersion of the surfactant PVP wrapped onto the CNTs in the epoxy matrix was observed. Usually, a higher content of CNTs in an epoxy can lead to reduction in the segmental motion and result in an agglomeration of nanoparticles in the polymer matrix. However, the wrapping process of PVP on the surface of the CNTs can effectively alleviate or avoid the agglomeration process through the interactions between the PVP molecules and the CNTs.

**Figure 10:**
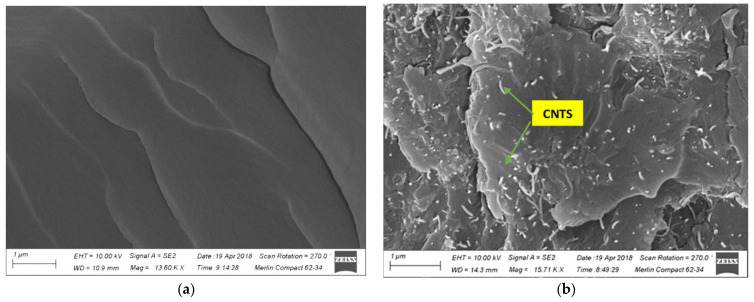

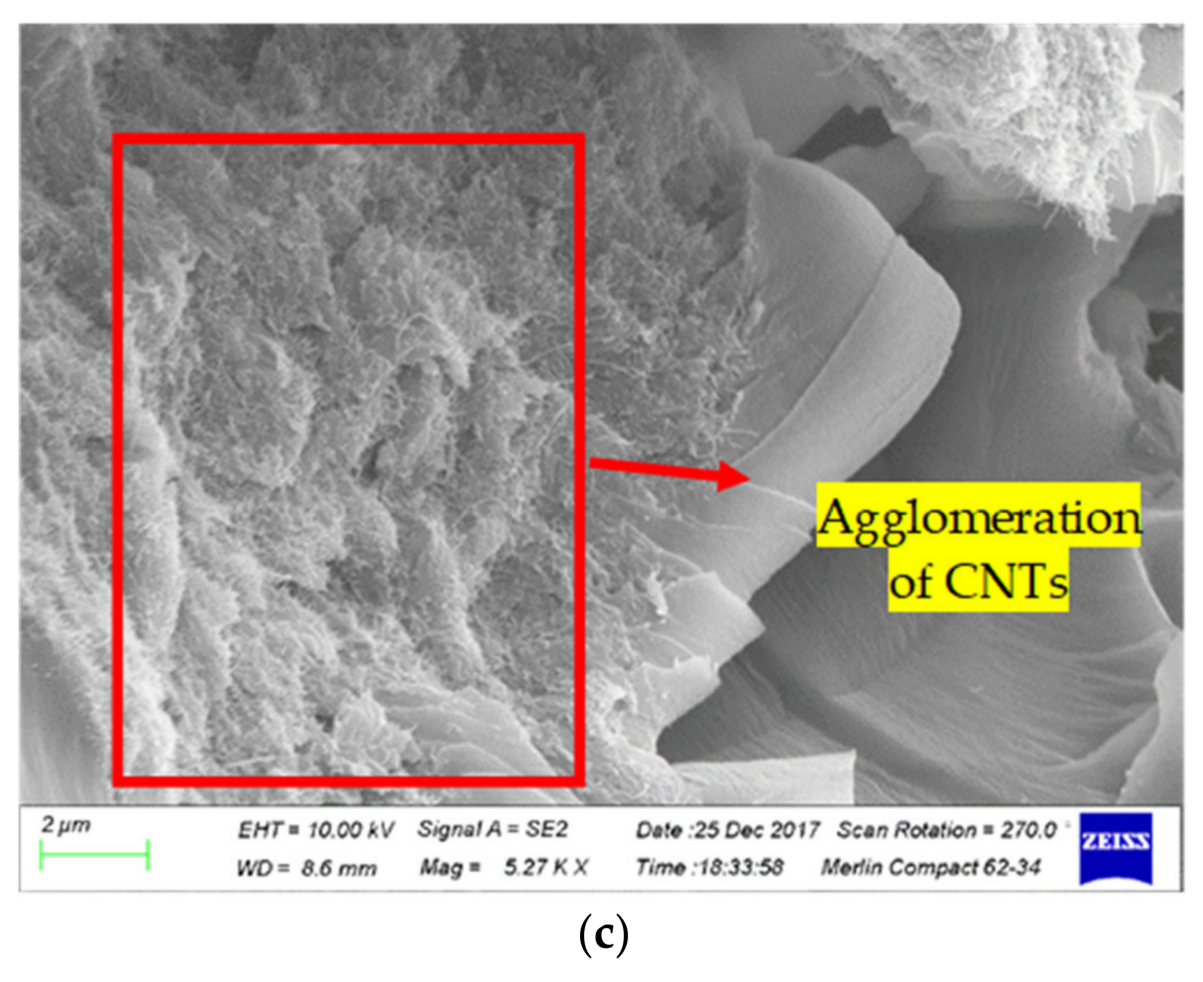
SEM images of epoxy/CNTs nanocomposites of (**a**) neat epoxy, (**b**) 1.5 wt.% of CNTs and (**c**) 2.0 wt.% of CNTs.

Figure 10 shows the fracture surface morphological analysis of the epoxy/CNT nanocomposites, including the neat epoxy (Figure 10a), their nanocomposites with the optimal ratio (1.5%) of CNTs, and the higher concentration (2.0%) of CNTs used in this study. Regarding Figure 10b, it can be observed that the dispersion of CNTs in the epoxy matrix was uniform at the optimal concentration. Figure 10c indicates the agglomeration of CNTs at a 2.0% weight concentration of PVP-modified CNTs in the epoxy matrix. After the process of mechanical mixing and sonication, the CNT-wrapped PVP can be effectively dispersed in the epoxy matrix. Moreover, the improvement in the mechanical and thermal properties of the nanocomposites occurred as shown in the above analysis. To compare, the fracture surface of the neat epoxy matrix was composed of long cracks with a relatively smooth surface, indicating the typical brittle fracture behavior.

### 3.4. Water Uptake Resistance

#### 3.4.1. Contact Angle Measurement

**Figure 11:**
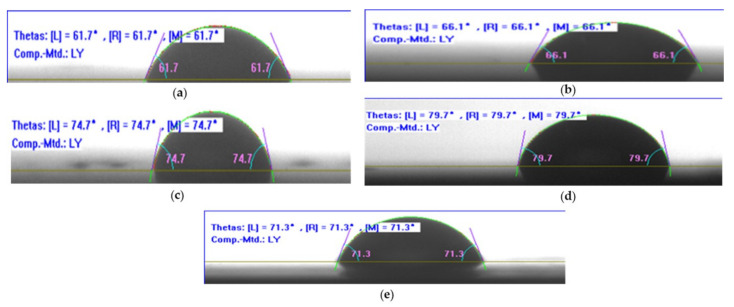
The contact angle images of (**a**) neat epoxy and (**b**) 0.5 wt.% of CNTs (**c**) 1 wt.% of CNTs (**d**) 1.5 wt.% (**e**) 2 wt.% of CNTs.

The contact angle of the neat epoxy and the epoxy/CNT nanocomposites were measured with deionized water droplets as shown in Figure 11. Evidently, the contact angle of the epoxy/CNT nanocomposite increased with the weight concentration of the CNTs. The contact angle, for example, was 61.7° for the neat epoxy and 79.7° for the nanocomposites with the 1.5 wt.% of CNTs. The incorporation of the CNTs into the epoxy could impart a reduction in the wettability on the surface of the epoxy/CNT nanocomposite compared to the neat epoxy [39]. The hydrophobicity of the epoxy/CNT nanocomposites was more prominent due to the presence of nanostructures, indicating a high surface energy [40]. Additionally, maximum hydrophobicity was observed when the weight concentration of the CNTs was 1.5%. Furthermore, a higher weight concentration of the CNTs led to the reduction of hydrophobicity due to the agglomeration of the CNTs in the epoxy matrix. 

#### 3.4.2. Water Absorption Behavior

**Figure 12:**
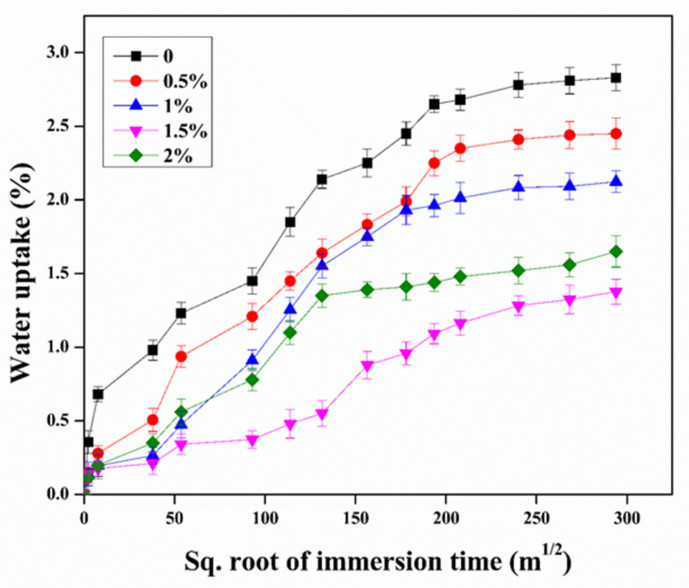
The water uptake versus square root of immersion time of epoxy/CNT nanocomposites.

The effect of the CNTs on the water uptake of the epoxy was investigated. Figure 12 shows the percentage of the water uptake curve versus the square root of the immersion time. Evidently, the water absorption curves show an initial increase and then a subsequent saturated condition. The water uptake rate largely decreased with the increase in the CNTs. A similar phenomenon was observed in the study [41], because the outstanding barrier effect of the CNTs might reduce the water uptake of the epoxy [27], which was consistent with the analysis results for the contact angle. Furthermore, the epoxy/CNT nanocomposites with the 1.5 wt.% of CNTs achieved the lowest water uptake compared to the other specimens, indicating the least hydrophilicity. Moreover, a higher added concentration (2%) of CNTs showed a greater water uptake owing to agglomeration possibly bringing about plenty of microcracks and microvoids in the epoxy [42]. To compare, the neat epoxy showed as more hydrophilic and obtained a higher amount of water uptake, owing to the hydrophilic nature of the epoxy. To summarize, the addition of the CNTs can obviously improve the hydrophobic behavior of the epoxy, which was significant in improving the durability of the epoxy exposed to the service conditions. 

## 4. Conclusions

Here, the mechanical and water uptake properties of epoxy nanocomposites with surfactant modified MWCNTs were investigated. The homogenous distribution of the CNTs in the epoxy matrix was achieved by wrapping the surfactant onto the CNTs. Thermal, mechanical, morphological, contact angle, and water uptake tests were performed to reveal the improvement mechanism of the CNTs on the epoxy matrix. The following conclusions can be drawn:

(1) The addition of CNTs into the epoxy caused a maximum increase percentage of 52.9% (flexural strength) and 25.5 % (flexural modulus), 29.5% (tensile strength) and 48.1% (tensile modulus) for the 1.5 wt.% of the CNTs. The improvement mechanism was attributed to strong interfacial bonding and an efficient load transfer between the epoxy and the CNTs. However, the mechanical properties of the epoxy/CNT nanocomposite decreased or saturated owing to the agglomeration effect when the weight concentration of the CNTs was 2.0%.
(2) The maximum increase percentage (~9.5%) of the glass transition temperature (Tg) and the lower height of the tan delta peak of the nanocomposites were achieved for the 1.5 wt.% of the CNTs compared to the neat epoxy matrix. This can be attributed to the uniform dispersion of the CNTs in the epoxy effectively improving the crosslinking between the carbon nanotubes and the epoxy, and a reduced free space of the macromolecular segmental motion. The higher addition ratio (2%) of CNTs caused agglomeration, which acted as a plasticizer and decreased the crosslinking and interfacial bonding between the epoxy matrix and the CNTs.
(3) The incorporation of the CNTs into the epoxy could impart a reduction in the wettability on the surface of the epoxy/CNT nanocomposite, leading to an increase in the contact angle and a reduction in the water uptake, which was significant to improve the durability of the epoxy. Moreover, a higher concentration (2%) of the CNTs showed a larger water uptake owing to the agglomeration possibly causing the formation of microcracks and microvoids in the epoxy.
(4) The wrapping of PVP onto the surface of the CNTs was confirmed by TEM analysis. A surface morphology analysis showed a uniform distribution of the surfactant-modified CNTs in the epoxy matrix formed at the weight concentration of 1.5%. Meanwhile, the agglomeration of the CNTs in the epoxy matrix was observed at a higher weight concentration of 2%. It is recommended that the weight concentration of PVP/CNTs in epoxy does not exceed the optimal concentration of 1.5% to ensure enhanced mechanical properties and a longer service life. A further investigation is needed to analyze the effect of PVP modified CNTs on the epoxy nanocomposite at a higher concentration (> 2%).

## Figures and Tables

**Table 1:** Increase percentage of flexural strength and modulus of epoxy/CNTs composites with the weight concentration of CNTs.

Weight Concentration of CNTs (%)	Flexural Strength (%)	Flexural Modulus (%)
0.0	/	/
0.5	11.1	5.6
1	30.3	10.5
1.5	52.9	25.5
2	42.8	25.8

**Table 2:** Increased percentage of tensile strength and modulus with the weight concentration of CNTs.

Weight of CNTs (%)	Tensile Strength (%)	Tensile Modulus (%)
Control	/	/
0.5	1.1	1.4
1	8.9	20.3
1.5	29.5	48.1
2	20.3	45.0

## Data Availability

Not applicable.
